# Sebaceous adenoma of the conjunctiva and caruncle: a clinicopathological report of three cases and literature review

**DOI:** 10.5935/0004-2749.20220023

**Published:** 2025-08-21

**Authors:** Mariana Borges Barcellos Dias, Melina Correia Morales, Arthur Gustavo Fernandes, Moacyr Rigueiro, Alexandre Nakao Odashiro, Rubens Mattos Belfort Neto

**Affiliations:** 1 Ocular Oncology Service, Universidade Federal de São Paulo, São Paulo, SP, Brazil; 2 Department of Ophthalmic Pathology, Universidade Federal de São Paulo, São Paulo, SP, Brazil; 3 Department of Ophthalmology and Pathology, Henry C. Witelson Ocular Pathology Laboratory & McGill University Health Centre, Montreal, QC, Canada

**Keywords:** Sebaceous gland neoplasm, Adenocarcinoma, Conjunctival neoplasms, Muir-Torre syndrome, Immunohistochemistry, Biopsy, Human, Case report, Neoplasia das glândula sebácea, Adenocarcinoma, Neoplasia da túnica conjuntiva, Síndrome de Muir-Torre, Imuno-Histoquímica, Biopsia, Humanos, Relato de caso

## Abstract

Sebaceous tumors of the conjunctiva and caruncle are rare conditions, accounting for 1% of caruncle lesions and even lower among conjunctival lesions. Almost 50% of cases are associated with Muir-Torre syndrome, a rare autosomal-dominant condition characterized by at least one sebaceous skin tumor and one visceral malignancy. We report 3 cases of sebaceous adenoma with different presentations that were submitted to excisional biopsy and immunohistochemical study. Diagnosis of these tumors should increase the level of suspicion and lead to clinical investigation to rule out neoplasms, particularly because in up to 41% of cases, these can be the first sign of the disease.

## Abstract (pt)

Tumores sebáceos da conjuntiva e carúncula são condições raras, sendo mais encontrados nas pálpebras. O adenoma sebáceo é responsável por 1% das lesões carunculares e ainda menos frequente nas lesões da conjuntiva. Esse diagnóstico é de suma importância pois quase 50% desses pacientes podem ser diagnosticados com a Síndrome de Muir-Torre, uma condição autossômica dominante rara que é caracterizada pela presença de ao menos um tumor de pele sebáceo e uma neoplasia visceral (gastrointestinal, câncer geniturinário e de mama). O estudo tem como objetivo relatar 3 casos de adenoma sebáceo com diferentes apresentações, que foram submetidos a biópsia excisional e estudo imuno-histoquímico. O diagnóstico desses tumores deve levantar suspeitas e aconselhar a investigação clínica para descartar outras neoplasias, principalmente porque em até 41% dos casos esse pode ser o primeiro sinal da Síndrome de Muir-Torre.

## INTRODUCTION

Sebaceous tumors of the conjunctiva and caruncle are rare conditions that are more frequently found in the eyelids. Sebaceous adenoma (SA) accounts for 1% of caruncle lesions^(1)^ and even lower among conjunctival lesions. To our best knowledge, only three cases of bulbar conjunctival SA have been reported in the literature^(2,3)^. The major importance of this diagnosis is that almost 50% of cases can be associated with Muir-Torre syndrome (MTS), a rare autosomal-dominant condition characterized by at least one sebaceous skin tumor and visceral malignancy such as gastrointestinal, genitourinary, and breast cancer^(4)^. In this article, we aimed to report 3 cases of SA with different presentations and, considering its rarity, raise awareness of this differential diagnosis and its correlation with other systemic tumors.

## CASE REPORTS

### Case 1

**Figure 1:**
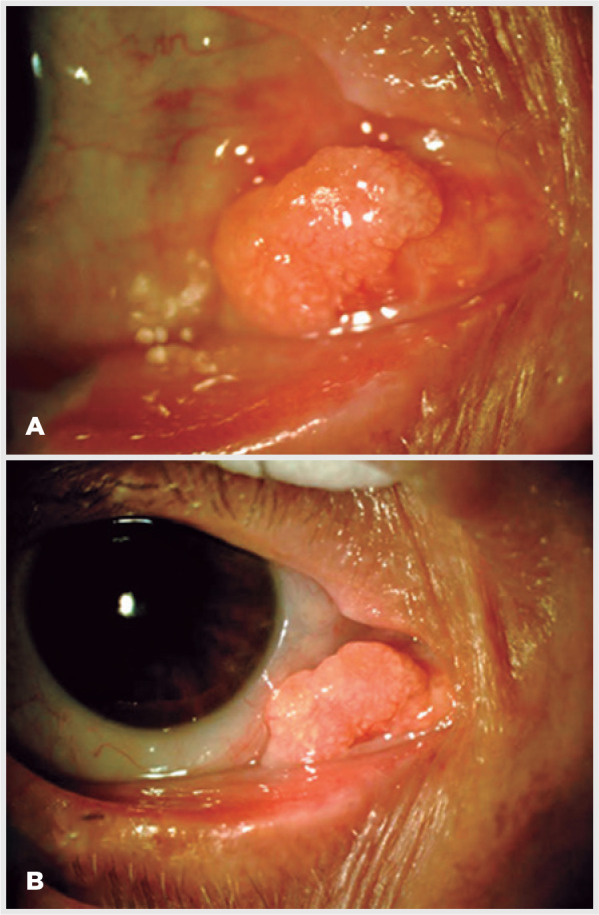
(A) Biomicroscopic examination image showing a yellowish conjunctival lesion, with a papillomatous aspect on plica semilunaris topography. (B) Higher magnification.

**Figure 2:**
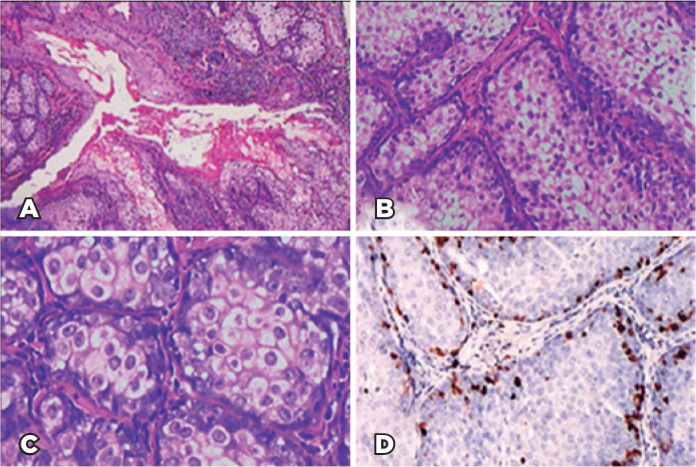
(A and B) Histopathological examination results showing a nodular and lobulated tumor (hematoxylin-eosin [H&E], staining, original magnifications ×40 and ×100). (C) At the center, the tumor is composed of mature sebocytes with a clear cytoplasm containing lipid vacuoles without atypia. The periphery of the lobules consisted of small darker cells represented by basaloid germinative cells (H&E, original magnification ×200). (D) Immunohistochemistry with Ki67, a cell proliferation marker, is mainly negative in well-differentiated sebaceous cells and positive at the periphery of the lobules in germinative cells. This is consistent with the diagnosis of sebaceous adenoma (SA).

**Figure 3:**
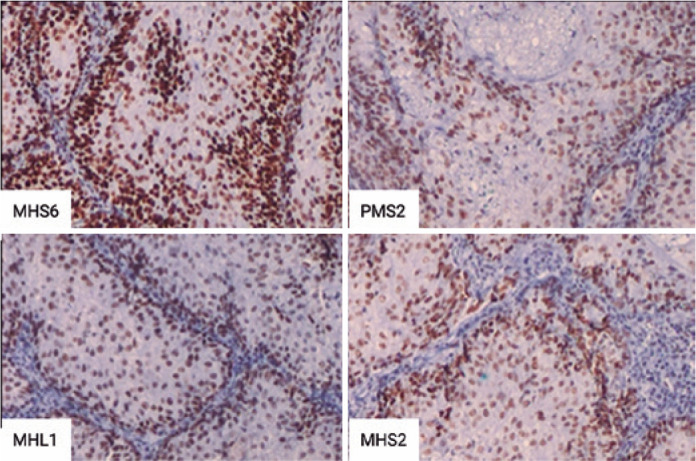
Immunohistochemistry of the mismatch repair proteins MSH6, PMS2, MHL1, and MSH2 showing nuclear positivity in the cells with all four markers.

A 73-year-old woman was referred to our service with a 6-month history of conjunctival lesion on her right eye. The best-corrected visual acuity (BCVA) was 20/40 in both eyes. Biomicroscopic examination revealed a yellowish conjunctival lesion on plica semilunaris topography, measuring approximately 4.6 mm in basal diameter, with no prominent conjunctival or episcleral vessels (Figure 1A and B). The left eye was normal. The patient underwent an excisional biopsy using the standard “no touch” technique. On the basis of the histopathological examination result, a diagnosis of SA was made (Figure 2). An immunohistochemical study for mismatch repair (MMR) proteins showed no abnormalities (Figure 3). No evidence of recurrence or systemic malignancy was found at 1-year follow-up.

### Case 2

**Figure 4:**
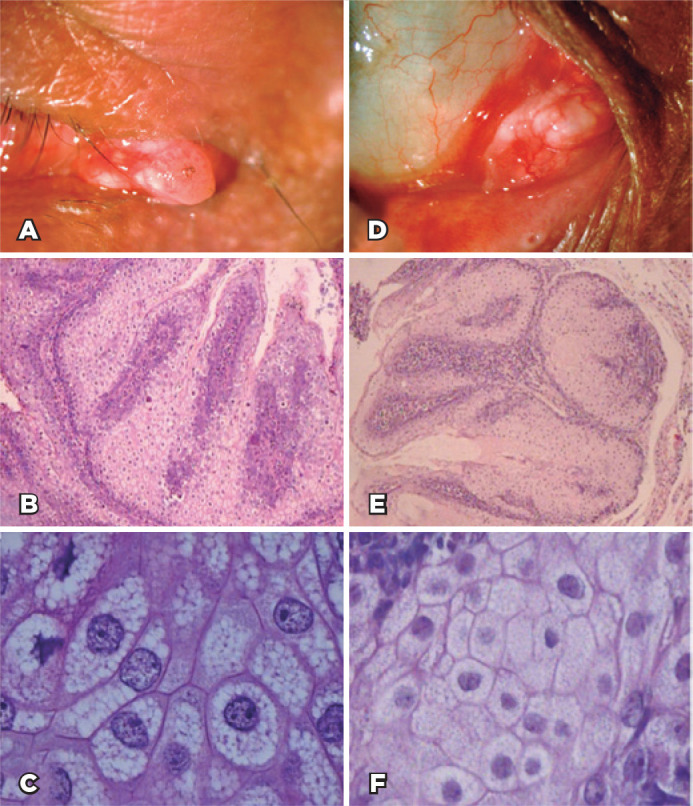
(A) Clinical appearance of a papillomatous caruncle nodule. (B) Low-power view showing a lobulated tumor composed of clear cells on the center and darker cells at the periphery of the lobules (hematoxylin-eosin staining [H&E], original magnification ×40). (C) The tumor is composed of mature sebocytes with clear cytoplasms without pleomorphism or brisk mitotic activity. At a higher magnification, the cells present round-to-oval nuclei with well-distributed chromatin, conspicuous nucleoli, and a vacuolated clear cytoplasm (H&E, original magnification ×400). (D) Yellowish subepithelial mass suggestive of sebaceous adenoma. (E) Histopathological examination result showing a well-delimitated and lobulated tumor, with features identical to those of cases 1 and 2 (H&E, original magnification ×40). (F) The clear cytoplasm represents empty spaces filled by the lipid vacuoles that were artefactually dissolved during specimen processing (H&E, original magnification ×400).

A 64-year-old man presented to our service with a complaint of a painless lesion in his right eye that had persisted for 7 months. The BCVA at the time of presentation was 20/20 in the right eye and 20/50 in the left eye. On slit-lamp biomicroscopy, a 4.0-mm pinkish elevated lesion was found on the caruncle region, with no dilated vessels on the conjunctiva (Figure 4A).

The left eye was normal. A malignant epithelial tumor was considered, and the patient underwent an excisional biopsy. The histopathological analysis confirmed the diagnosis of SA with free margins (Figure 4B and C). No signs of recurrence or systemic malignancies were detected during the 6-month follow-up.

### Case 3

A 58-year-old woman with a complaint of watery right eye for 3 years was referred to our service. A nodular amelanotic mass was observed on caruncle topography. She had a smoking history of 28 pack-years and past medical history consisting of breast cancer treated with surgery and radiotherapy 2.5 years before, with good systemic control. Her BCVA was 20/20 on both eyes. Ocular examination revealed a 10-mm amelanotic yellowish mass in the caruncle of the right eye and no abnormality in the left eye (Figure 4D). The patient underwent a “no touch” excisional biopsy. The histopathological examination revealed a tumor with features identical to those of the two previous cases (Figure 4E and F). An immunohistochemical study for MMR proteins showed no abnormalities.

## DISCUSSION

The caruncle is located in the medial canthus of the eye and contains both cutaneous and conjunctival structures such as hair follicles and sebaceous glands. On the other hand, the plica semilunaris does not contain sebaceous glands, consisting only of conjunctival structures, which explains the singularity of sebaceous tumors in this location.

A previously published survey among 191 patients with caruncle lesions^(1)^ reported only 2 patients with SA. In older reviews, the incidence was even lower^(5,6)^. Meanwhile, only three cases of primary bulbar conjunctival SA have been reported in the literature^(2,3)^.

In our case series, all the patients presented with yellowish elevated lesions with no prominent feeder vessels, which is consistent with descriptions in the literature^(1)^. Tumors with this appearance should be considered in the differential diagnosis of amelanotic nodular tumors in the conjunctiva and caruncle, such as conjunctival carcinoma and amelanotic melanoma. As the differentiation can only be made by histopathological analysis, biopsy should always be performed.

MTS is an autosomal-dominant disorder characterized by the presence of sebaceous tumors and visceral malignancies, mostly colorectal, genitourinary, and breast cancers^(4,7)^. Among the sebaceous tumors, SA and “sebaceous carcinoma” are the two entities most related to MTS. Diagnosis of these tumors should increase the level of suspicion and lead to clinical investigation to rule out neoplasms, particularly because in up to 41% of cases, these can be the first sign of the disease^(4)^.

MTS is caused by germline mutations in one allele of the DNA MMR genes MLH1, MSH2, and MSH6. The MMR system consists of human mutS homolog 2 (hMSH2), human mutS homolog 3 (hMSH3), human mutS homolog 6 (hMSH6), human mutL homolog 1 (hMLH1), and human post-meiotic segregation increased 2 (hPMS2) proteins. They are responsible for maintaining genomic integrity by correcting base substitution and small insertion-deletion mismatches generated by errors in base pairing during DNA replication. Microsatellite instability (MSI) is the hallmark of MMR gene deficiency. The loss of DNA MMR function due to germline and/or somatic inactivating mutations of MMR genes leads to the accumulation of mutations across the genome and mainly in the microsatellite repetitive sequences, creating a molecular phenotype known as MSI. Historically, the diagnosis of MTS is purely clinical, but owing to improvement in the genetic basis of the disease, this reality may change soon^(8)^. In this scenario, immunohistochemical analysis of the tumors can be of great value because the pathogenic variants of the MMR genes associated with MTS can be indirectly assessed by immunohistochemistry, a rapid and inexpensive method of analysis^(9)^. The most common MMR protein deficiency in MTS is MSH2 followed by MLH1^(7)^. Although none of our patients presented with abnormal MMR expressions, patient three had a history of breast carcinoma, which increased the suspicion of MTS.

It is prudent to test all SAs and skin sebaceous tumors for MMR, given the low incidence of this tumor in the general population and their frequent association with MTS^(10)^.
